# Azithromycin alleviates systemic lupus erythematosus via the promotion of M2 polarisation in lupus mice

**DOI:** 10.1038/s41420-021-00466-4

**Published:** 2021-04-16

**Authors:** Jie Wang, Qian Chen, Zhixiong Zhang, Shangshang Wang, Yilun Wang, Mengmeng Xiang, Jun Liang, Jinhua Xu

**Affiliations:** Department of Dermatology, Huashan Hospital, Fudan University, Shanghai, China

**Keywords:** Drug discovery, Immunological disorders

## Abstract

Our previous study demonstrated that azithromycin could promote alternatively activated (M2) macrophages under lupus conditions in vitro, which might be beneficial for lupus treatment. Thus, the aim of this study was to further confirm whether azithromycin can drive M2 polarisation in lupus and ultimately alleviate systemic lupus erythematosus (SLE) in vivo. Lymphocyte-derived DNA (ALD-DNA)-induced mice (induced lupus model) and MRL-*Fas*^lpr^ mice (spontaneous lupus model) were both used in the experiment. First, we observed symptoms of lupus by assessing the levels of serum anti-dsDNA antibodies and serum creatinine and renal pathology. We found that both murine models showed increased levels of serum anti-dsDNA antibodies and creatinine, enhanced glomerular fibrosis and cell infiltration, basement membrane thickening and elevated IgG deposition. After azithromycin treatment, all these medical indexes were alleviated, and kidney damage was effectively reversed. Next, macrophage polarisation was assessed in the spleen and kidneys. Macrophage infiltration in the spleen was notably decreased after azithromycin treatment in both murine models, with a remarkably elevated proportion of M2 macrophages. In addition, the expression of interleukin (IL)-1, IL-6, tumour necrosis factor (TNF)-α, inducible nitric oxide synthase (iNOS), CD86, toll-like receptor (TLR)2 and TLR4 was extremely downregulated, while the expression of transforming growth factor (TGF)-β, arginase-1 (Arg-1), chitinase-like 3 (Ym-1), found in inflammatory zone (Fizz-1) and mannose receptor (CD206) was significantly upregulated in the kidneys after azithromycin treatment. Taken together, our results indicated for the first time that azithromycin could alleviate lupus by promoting M2 polarisation in vivo. These findings exploited the newly discovered potential of azithromycin, a conventional drug with verified safety, affordability and global availability, which could be a novel treat-to-target strategy for SLE via macrophage modulation.

## Introduction

Systemic lupus erythematosus (SLE) is a chronic autoimmune disease affecting multiple systems and leading to a broad spectrum of manifestations ranging from mild to fatal. In 2015, Wang et al.^1^ reported that infections, renal involvement, lupus encephalopathy and cardiovascular disease were the leading causes of death from SLE in China. Despite great progress over the past few decades, the mortality rate remains high compared with that of the non-SLE population, and SLE is still the leading cause of death in young females^2–5^. Explorations focused on the treatment of SLE are still underway.

Currently, most treatments for SLE are conventional agents that are not target-specific, which results in unexpected adverse effects and even premature mortality in SLE patients^6^. Nowadays, with a deeper understanding of SLE pathogenesis, treat-to-target therapies are being developed. Belimumab is now the only targeted biologic agent approved for SLE, which inhibits B-cell activating factor^7^. However, the cost of belimumab hampers its use, and nearly one-third of patients were not sensitive to belimumab treatment in a clinical trial^8^. Therefore, it is imperative to explore more effective and feasible therapies for SLE.

To date, hundreds of registered studies have tested therapies for SLE targeting B cells, long-lived plasma cells, CD22, CD40-CD40 ligand, etc. However, the role of macrophages in SLE has not been fully elucidated and has drawn our attention. Activated macrophages are categorised into two main subsets: the proinflammatory subset (classically activated macrophages, M1) and the anti-inflammatory subset (alternatively activated macrophages, M2)^9,10^. Recent studies have highlighted the imbalance in macrophage polarisation between the M1 and M2 phenotypes in SLE. Compared with those from healthy controls, monocyte-derived macrophages (MDMs) from SLE patients triggered by apoptotic cells show an increased proinflammatory (M1-like) profile^11^. During outbreaks of lupus nephritis in SLE model mice, macrophages in the kidneys are dramatically skewed toward the M1 phenotype rather than the M2 phenotype^12,13^. Thus, therapies targeting macrophage polarisation are promising for SLE treatment.

Azithromycin (AZM) is a broad-spectrum antibiotic that has been manufactured and used globally for nearly half a century. AZM has been verified to have a long half-life and good record of safety and is consistently being developed for new uses, including as a potential candidate treatment for Corona Virus Disease (COVID)-19 (refs. ^14,15^). A growing number of studies have shown that AZM can improve cystic fibrosis, spinal cord injury and pulmonary diseases by promoting M2 polarisation^16–20^. Therefore, we hypothesised that AZM can alleviate SLE by driving M2 polarisation. Our previous study demonstrated that AZM could promote an alternatively activated macrophage phenotype in SLE in vitro via the PI3K/Akt signalling pathway^21^. However, whether AZM can induce M2 polarisation in lupus in vivo, which in turn would lead to alleviation, urgently needs to be confirmed.

In this study, lymphocyte-derived DNA (ALD-DNA)-induced mice were used to establish an in vivo SLE model as previously described^22–24^. MRL-*Fas*^lpr^ mice, in which autoimmune disease develops spontaneously, steadily develop disease similar to human lupus and were also utilised in our experiment^25,26^. The aim of the study was to verify whether AZM can relieve SLE via macrophage modulation, gaining deeper insights into the role of AZM in SLE alleviation.

## Results

### Azithromycin alleviated the symptoms of ALD-DNA-induced murine lupus

**Fig. 1:**
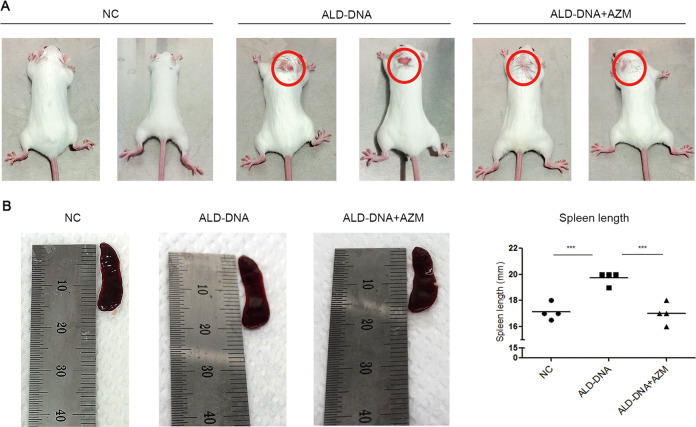
Morphologic characterisation of the fur and spleen of mice. **A** Obvious patchy hair loss emerged around the injection site in the ALD-DNA group, while the mice treated with azithromycin showed distinct hair regeneration. **B** Compared with the normal control group, the ALD-DNA group showed a longer spleen length, and spleen length was restored in the azithromycin group. *N* = 4. ***p < 0.001.

Subcutaneous injection of ALD-DNA at an initial time point was used to induce murine lupus as described previously^22–24^. Four weeks after immunisation, hair loss started to occur around the injection site in the ALD-DNA group and peaked with obvious patchy hair loss 12 weeks later. Interestingly, after two weekly intraperitoneal injections of AZM, treated mice showed distinct hair regeneration (Fig. 1A). In addition, the spleen length was longer in the ALD-DNA group than in the normal control group (20.0 ± 0.5 mm *vs*. 17.0 ± 0.5 mm, *p* = 0.000) and was restored in the AZM treatment group (17.0 ± 1.0 mm vs. 20.0 ± 0.5 mm, *p* = 0.000; Fig. 1B).

**Fig. 2:**
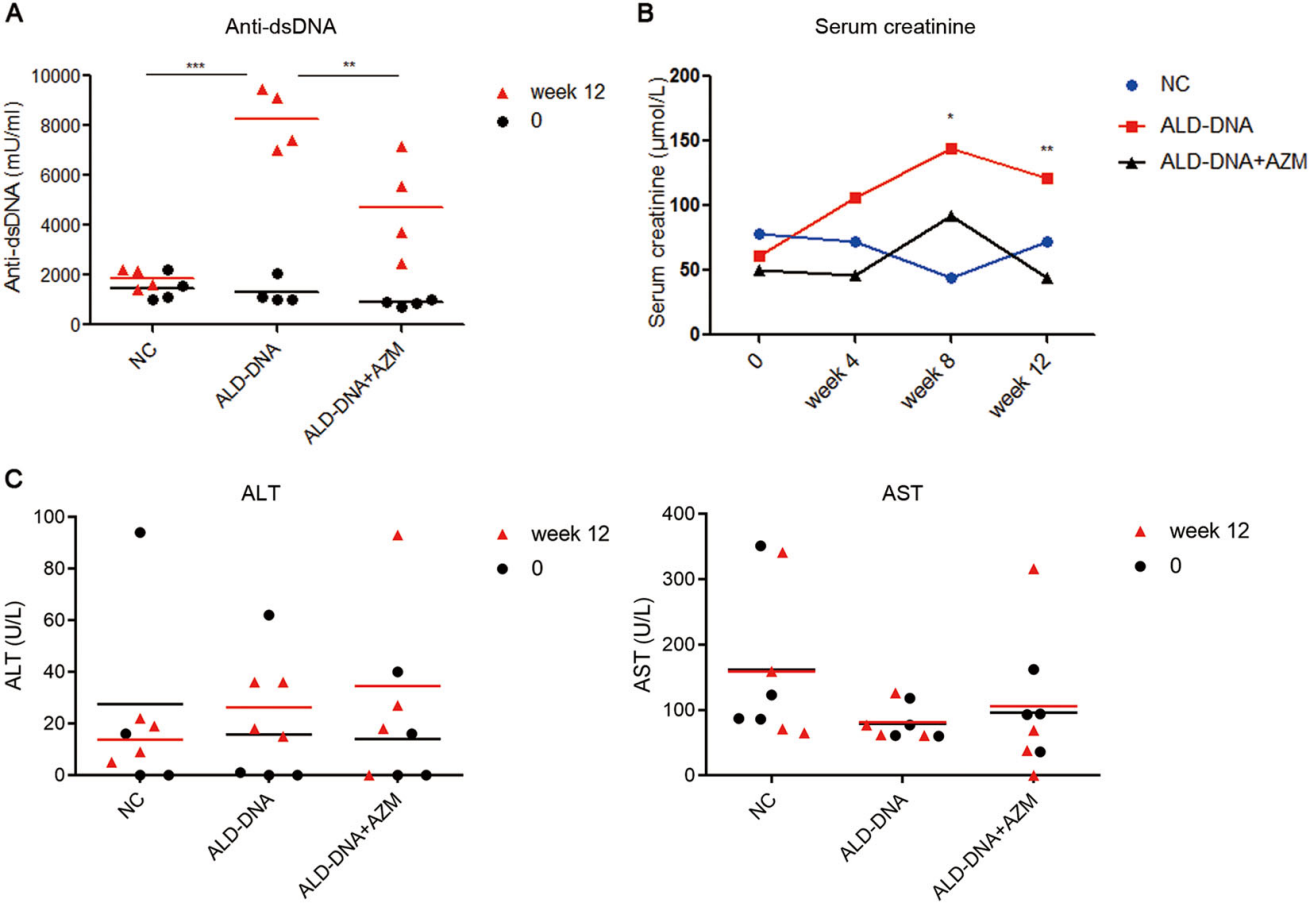
Azithromycin alleviated the serum levels of anti-dsDNA antibodies and creatinine in ALD-DNA-induced murine lupus in vivo. **A**.The serum level of anti-dsDNA antibodies was extremely higher in the ALD-DNA group than in the normal control group. The level was decreased after azithromycin treatment but was still higher than that in the normal controls. **B** The level of serum creatinine was largely increased and peaked at the 8th week in the ALD-DNA group but only slightly elevated in the azithromycin group. **C** There were no significant changes in ALT or AST among all groups. *N* = 4, *p < 0.05, **p < 0.01, ***p < 0.001.

For autoimmune antibodies, the serum level of anti-dsDNA antibodies was extremely elevated in the ALD-DNA group (8 235.00 ± 1 214.27 mU/ml, *p* = 0.000). However, there was a decrease in serum anti-dsDNA antibody levels after AZM treatment (4714.00 ± 2048.51 mU/ml, *p* = 0.006; Fig. 2A). As an indicator of kidney function, serum creatinine levels were largely increased and peaked at 143.75 ± 10.21 μmol/L after ALD-DNA immunisation but were only slightly elevated in the AZM group, with a peak of 91.75 ± 33.18 μmol/L (Fig. 2B). No significant changes were observed across all groups in alanine aminotransferase (ALT) or aspartate aminotransferase (AST; Fig. 2C).

**Fig. 3:**
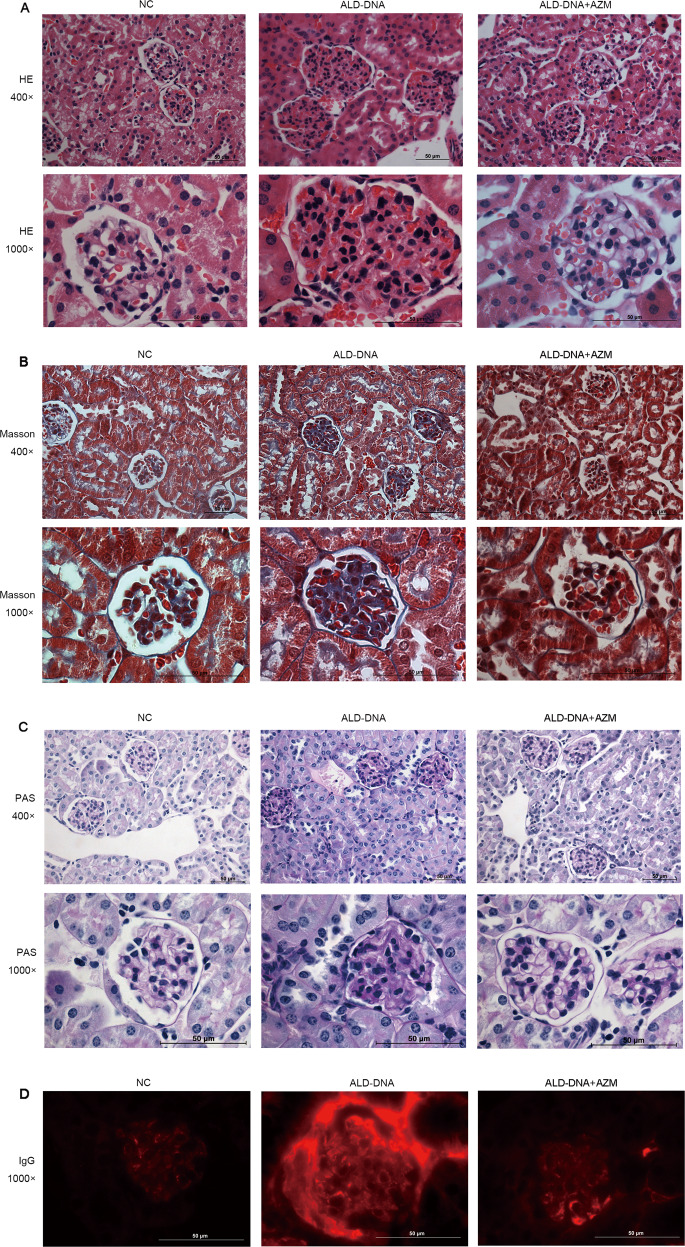
Azithromycin effectively reversed kidney damage in ALD-DNA-induced murine lupus in vivo. **A**–**C** Renal pathology was evaluated by haematoxylin and eosin (**A**), Masson (**B**) and periodic acid–Schiff (**C**) staining of renal tissues, and all showed significant remission after azithromycin treatment. **D** There was more IgG deposition in the ALD-DNA group than in the azithromycin group. *N* = 4. Scale bar = 50 μm.

In the kidneys, haematoxylin and eosin (H&E) staining of representative renal sections from the ALD-DNA group identified swelling of the renal tubules and glomeruli accompanied by substantial inflammatory cell infiltration. All of these changes were dramatically tempered in the AZM group (Fig. 3A). Masson staining analysis revealed increased interstitial fibrosis and nearly complete glomerulosclerosis in the ALD-DNA group, which were significantly mitigated after AZM treatment (Fig. 3B). In addition, the capillary loops of the glomerulus were well defined and thin in the AZM group and normal control group, while the mesangial cells and matrix were hyperproliferative in the ALD-DNA group, as represented by periodic acid–Schiff (PAS) staining (Fig. 3C). Additionally, the ALD-DNA group had much more IgG deposition in the glomerulus than the AZM group (Fig. 3D).

### Azithromycin drives M2 polarisation in ALD-DNA-induced mice

**Fig. 4:**
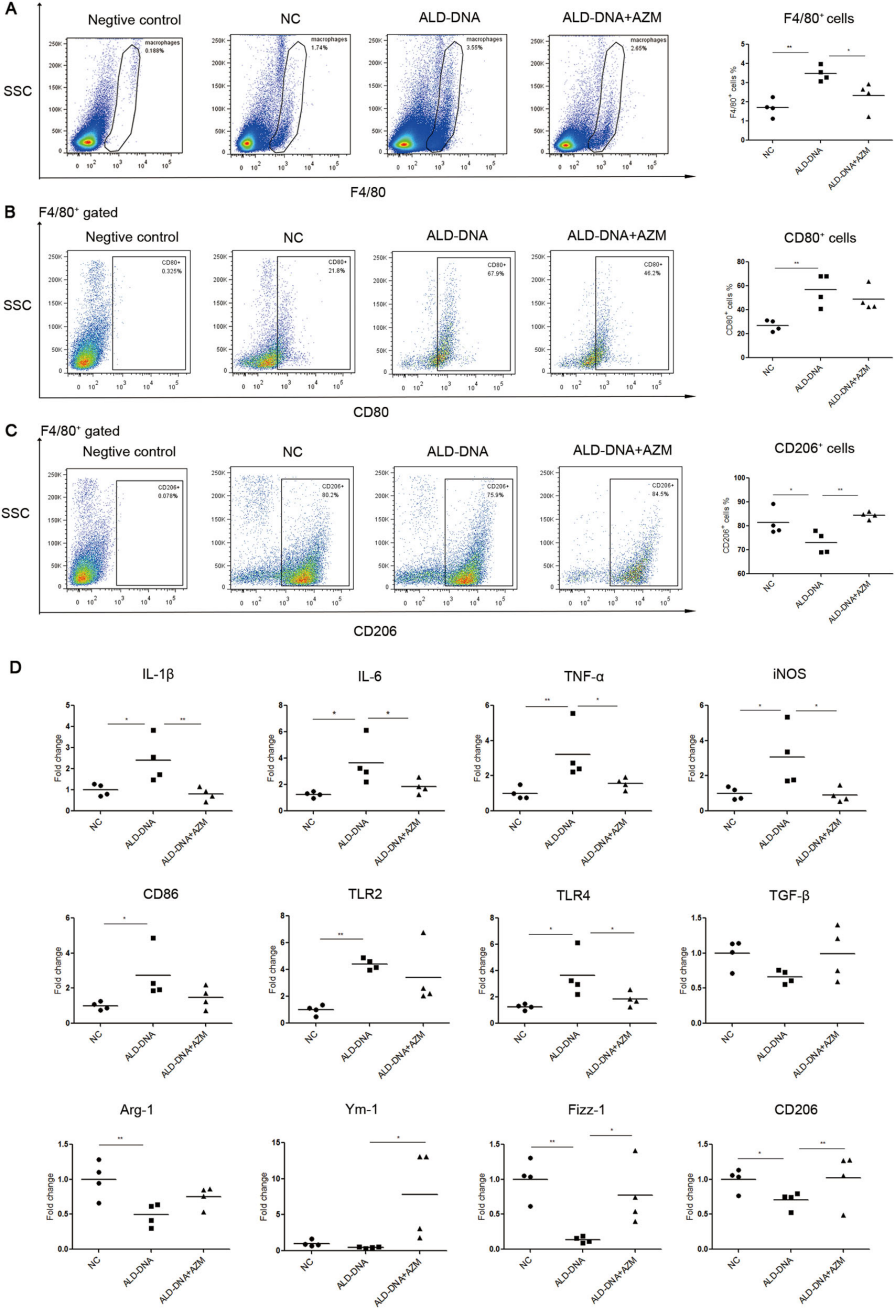
Azithromycin promoted M2 polarisation in ALD-DNA-induced murine lupus in vivo. **A** Macrophage infiltration was significantly increased in the ALD-DNA group but decreased after azithromycin treatment. **B**, **C** The proportion of F4/80^+^CD80^+^ cells (M1) was increased in the ALD-DNA group compared with the normal control group and reduced after azithromycin treatment (**B**), while the proportion of F4/80^+^CD206^+^ cells (M2) was decreased in the ALD-DNA group but rebounded with azithromycin treatment (**C**). **D** Azithromycin downregulated the increased expression of IL-1β, IL-6, TNF-α, iNOS, CD86, TLR2 and TLR4 observed in the ALD-DNA group and upregulated the decreased expression of TGF-β, Arg-1, Ym-1, Fizz-1 and CD206 observed in the ALD-DNA group. *N* = 4. Negative control: the unstained cells. *p < 0.05, **p < 0.01.

Spleens were analysed 12 weeks after immunisation. ALD-DNA significantly increased macrophage infiltration (3.48 ± 0.34%, *p* = 0.001), while AZM decreased macrophage infiltration (2.32 ± 0.75%, *p* = 0.015; Fig. 4A). In the spleen, the M1 population (F4/80^+^CD80^+^) of macrophages was remarkably increased upon ALD-DNA immunisation (56.88 ± 13.47%, *p* = 0.002), while the proportion was reduced to 50.77 ± 11.36% after AZM treatment (Fig. 4B). Correspondingly, ALD-DNA reduced the M2 population (F4/80^+^CD206^+^) of macrophages to 73.03 ± 4.61% (*p* = 0.028), while the proportion remained at 83.93 ± 1.25% (*p* = 0.004) in the AZM group (Fig. 4C).

To further investigate macrophage function, we compared the expression of genes associated with M1 and M2 macrophages in the kidney, which was reported previously^27–29^. Following ALD-DNA immunisation, the levels of M1 markers such as interleukin (IL)-1β (2.39 ± 1.06-fold change, *p* = 0.015), IL-6 (3.64 ± 1.72-fold change, *p* = 0.010), tumour necrosis factor (TNF)-α (3.23 ± 1.57-fold change, *p* = 0.009), inducible nitric oxide synthase (iNOS) (3.05 ± 1.71-fold change, *p* = 0.021), CD86 (2.74 ± 1.43-fold change, *p* = 0.025), toll-like receptor (TLR)2 (4.42 ± 0.41-fold change, *p* = 0.006) and TLR4 (3.64 ± 1.72-fold change, *p* = 0.010) were substantially elevated in the kidneys, while the levels of M2 markers such as transforming growth factor (TGF)-β (0.67 ± 0.10-fold change, *p* = 0.096), arginase-1 (Arg-1) (0.49 ± 0.16-fold change, *p* = 0.006), chitinase-like 3 (Ym-1) (0.45 ± 0.10-fold change, *p* = 0.833), found in inflammatory zone (Fizz-1; 0.14 ± 0.04-fold change, *p* = 0.003) and mannose receptor (CD206; 0.70 ± 0.16-fold change, *p* = 0.017) showed significant declining trends compared to those observed following control treatment. In contrast, intraperitoneal injection of AZM caused decreased levels of IL-1β (0.80 ± 0.31 vs. 2.39 ± 1.06-fold change, *p* = 0.007), IL-6 (1.84 ± 0.55 vs. 3.64 ± 1.72-fold change, *p* = 0.038), TNF-α (1.57 ± 0.32 vs. 3.23 ± 1.57-fold change, *p* = 0.035), iNOS (0.90 ± 0.41 vs. 3.05 ± 1.71-fold change, *p* = 0.017), CD86 (1.46 ± 0.64 vs. 2.74 ± 1.43-fold change, *p* = 0.079), TLR2 (3.40 ± 2.27 vs. 4.42 ± 0.41-fold change, *p* = 0.314) and TLR4 (1.84 ± 0.55 vs. 3.64 ± 1.72-fold change, *p* = 0.038) and increased levels of TGF-β (0.99 ± 0.38 vs. 0.67 ± 0.10-fold change, *p* = 0.106), Arg-1 (0.75 ± 1.49 vs. 0.49 ± 0.16-fold change, *p* = 0.100), Ym-1 (7.78 ± 6.15 vs. 0.45 ± 0.10-fold change, *p* = 0.017), Fizz-1 (0.77 ± 0.45 vs. 0.14 ± 0.04-fold change, *p* = 0.017) and CD206 (1.20 ± 0.13 vs. 0.70 ± 0.16-fold change, *p* = 0.002) compared with ALD-DNA immunisation alone (Fig. 4D).

### Azithromycin alleviated symptoms of lupus in MRL-*Fas*^lpr^ mice by polarising macrophages to the M2 phenotype

**Fig. 5:**
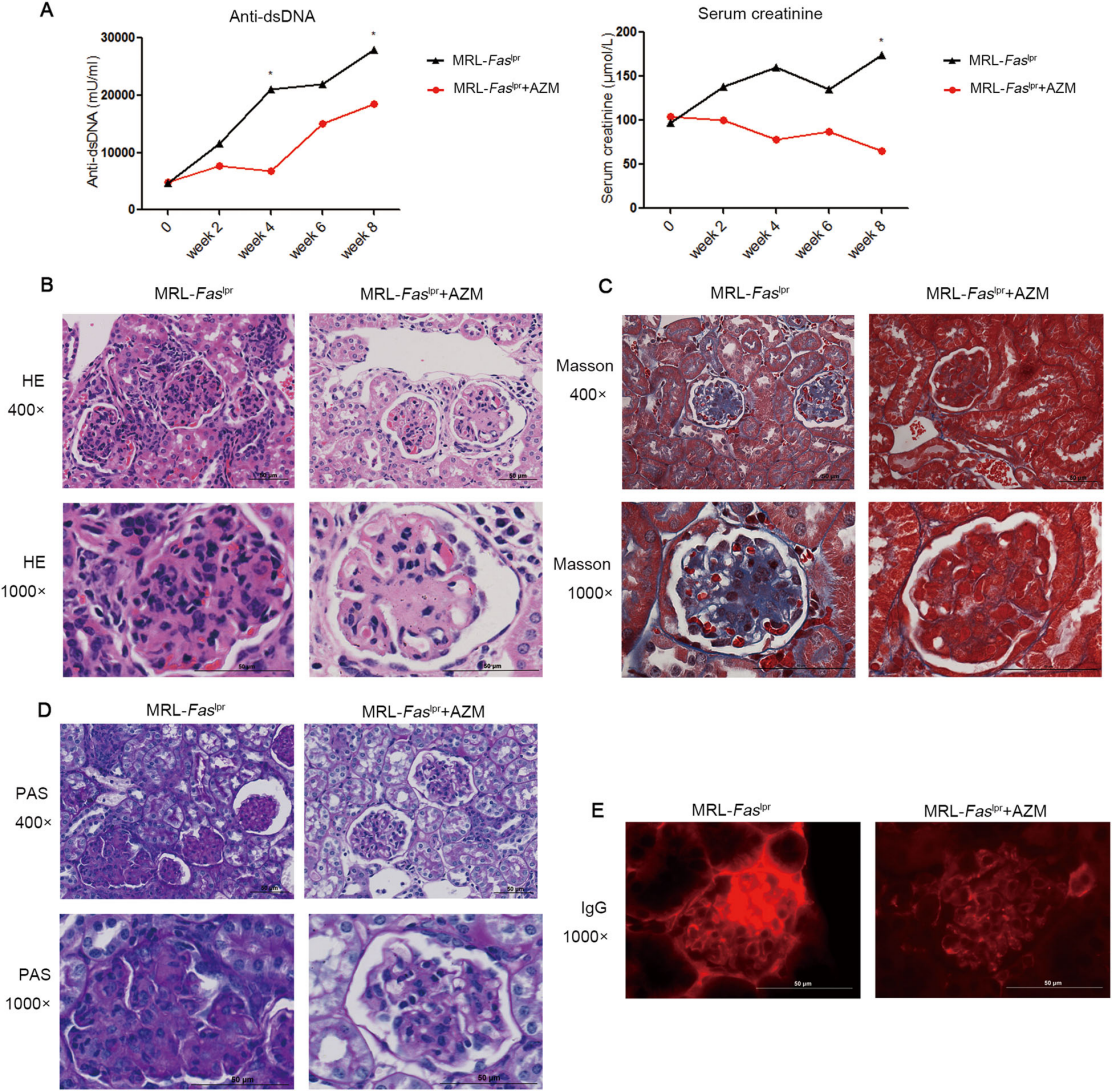
Azithromycin alleviated symptoms of lupus in MRL-*Fas*^lpr^ mice in vivo. **A** The azithromycin group showed a slowdown in the upward trend of the anti-dsDNA antibody level and a decline in the serum creatinine level. **B**–**E** Renal pathology evaluated by haematoxylin and eosin (**B**), Masson (**C**) and periodic acid–Schiff (**D**) staining and IgG deposition (**E**) showed remarkable mitigation of kidney damage after azithromycin treatment. *N* = 4. Scale bar = 50 μm.

For further evaluation of AZM efficacy, we next tested the role of AZM in vivo in MRL-*Fas*^lpr^ mice. As a lupus-prone mouse model, MRL-*Fas*^lpr^ mice showed elevated levels of anti-dsDNA antibodies (27,929.48 ± 2626.56 mU/ml, *p* = 0.038) and serum creatinine (174.33 ± 81.31 μmol/L, *p* = 0.020) at the end point. However, after AZM treatment, there was a slowdown in the upward trend of anti-dsDNA antibody levels, with a maximum of 18 439.23 ± 4699.32 mU/ml, and the level of serum creatinine was extremely decreased (Fig. 5A). To confirm whether the serological status reflected pathologic findings, we examined mouse kidneys microscopically. We found that MRL-*Fas*^lpr^ mice suffered great renal damage characterised by relatively high inflammatory infiltration, renal tubular swelling, interstitial congestion, glomerulofibrosis, IgG deposition and glomerular basement membrane thickening. As expected, AZM effectively reversed this kidney damage (Fig. 5B–E).

**Fig. 6:**
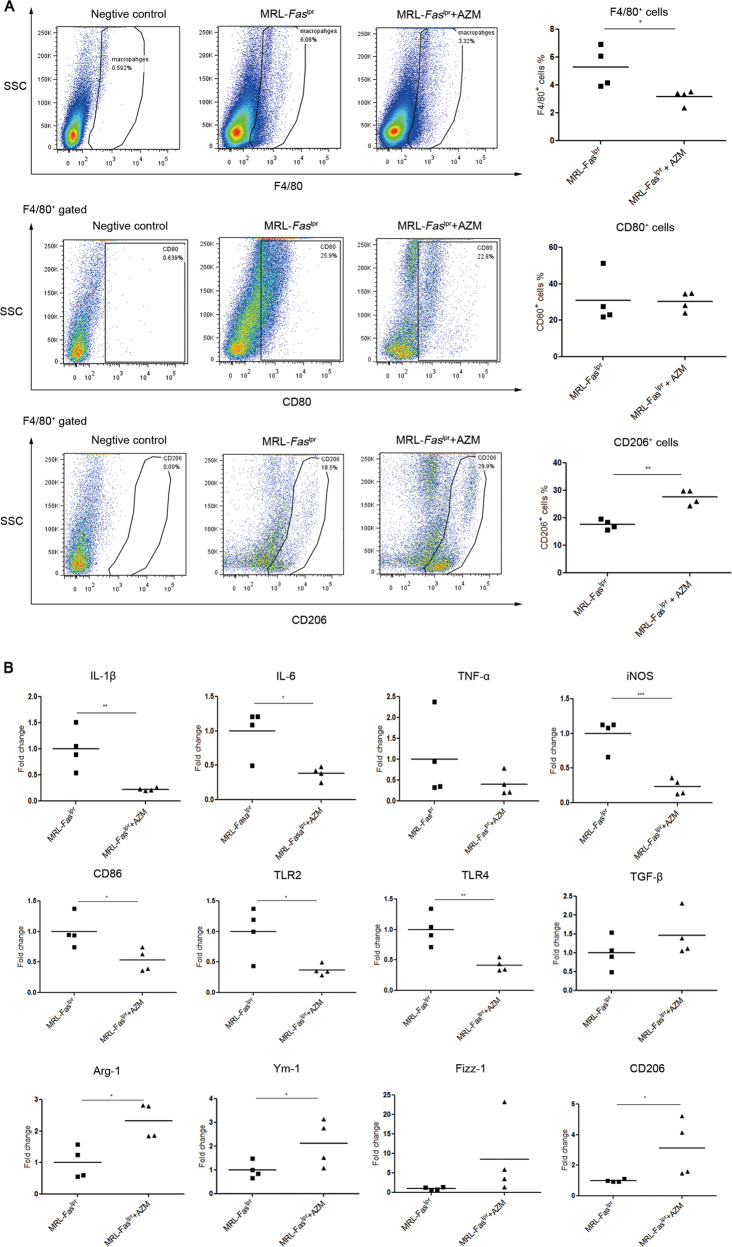
Azithromycin promoted M2 polarisation in MRL-*Fas*^lpr^ mice in vivo. **A** After azithromycin treatment, macrophage infiltration in the spleen was markedly decreased, and the M1 proportion showed no significant change, but the M2 proportion was notably increased. **B** The expression of M1-related genes including IL-1β, IL-6, TNF-α, iNOS, CD86, TLR2 and TLR4 was decreased, while that of M2-related genes including TGF-β, Arg-1, Ym-1, Fizz-1 and CD206 was increased post azithromycin treatment. *N* = 4. Negative control: the unstained cells. *p < 0.05, **p < 0.01.

Next, we examined the impact of AZM on macrophage polarisation in MRL-*Fas*^lpr^ mice. The total number of macrophages infiltrated in the spleen was markedly decreased after AZM treatment (3.15 ± 0.53% vs. 5.27 ± 1.47%, *p* = 0.035). Although the proportion of M1 macrophages exhibited no significant difference between the two groups (30.93 ± 13.80% vs. 30.33 ± 5.22%, *p* = 0.938), the proportion of M2 macrophages was extremely higher in the AZM group (27.55 ± 2.79% vs. 17.65 ± 1.81%, *p* = 0.001; Fig. 6A). Additionally, this M2 polarisation was confirmed by the markedly elevated expression levels of M2-related genes such as TGF-β (1.47 ± 0.59-fold change, *p* = 0.246), Arg-1 (2.33 ± 0.55-fold change, *p* = 0.012), Ym-1 (2.13 ± 0.98-fold change, *p* = 0.037), Fizz-1 (8.53 ± 10.01-fold change, *p* = 0.183) and CD206 (3.11 ± 1.87-fold change, *p* = 0.033) and the reduced expression levels of M1-related genes including IL-1β (0.23 ± 0.03-fold change, *p* = 0.008), IL-6 (0.38 ± 0.10-fold change, *p* = 0.013), TNF-α (0.40 ± 0.27-fold change, *p* = 0.280), iNOS (0.23 ± 0.12-fold change, *p* = 0.0009), CD86 (0.53 ± 0.19-fold change, *p* = 0.027), TLR2 (0.37 ± 0.09-fold change, *p* = 0.023) and TLR4 (0.42 ± 0.09-fold change, *p* = 0.006) in the kidneys from the AZM group (Fig. 6B).

## Discussion

In recent years, accumulating evidence has suggested that M2 polarisation contributes to lupus remission^30–32^. Our previous study also confirmed that AZM could dampen inflammatory reactions by promoting M2 polarisation in imitated lupus conditions in vitro^21^. However, whether AZM can relieve lupus in vivo remained unknown. Thus, we further investigated the effect of AZM on lupus mice in vivo.

First, to be consistent with what we published previously, BALB/c mice were subcutaneously immunised with ALD-DNA to generate a lupus model. The ALD-DNA lupus mouse model was first established by Qiao and has since been applied in several lupus-related studies^33,34^. We found no significant difference in serum anti-dsDNA antibody levels among all groups at the initial time point. However, the level of serum anti-dsDNA antibodies was extremely elevated at 12 weeks after ALD-DNA immunisation in the ALD-DNA group, which was consistent with the previous observation by Qiao et al. As serum anti-dsDNA antibodies are the specific autoantibodies in SLE, ALD-DNA resulted in autoantibody changes that fit well with those in SLE. Notably, while AZM treatment was administered at the same time as ALD-DNA immunisation, the level of serum anti-dsDNA antibodies was significantly decreased, which indicated that AZM successfully inhibited the production of autoantibodies in lupus. However, the level of serum anti-dsDNA antibodies was still higher than that in normal controls. In our opinion, this might be due to the dosing strategy. Thus, further studies are needed to improve the efficacy in terms of dosage, delivery route and length of administration. Additionally, spleens from mice in the ALD-DNA group were enlarged compared with those from normal controls, in line with excessive autoimmunity. As expected, spleen size decreased after AZM treatment. Unexpectedly, there was an extreme loss of hair around the injection site, and we speculated that this might be due to attack on the hair follicles by overactivated T cells induced by ALD-DNA^35^. Interestingly, the hairs regrew after AZM treatment; we speculated that this was due to the AZM-induced M2 polarisation, as Szu-Ying Chu previously reported M2 macrophages are beneficial to hair regeneration^36^.

The kidneys are known to be highly susceptible to SLE. Even with no clinical symptoms of renal damage, almost 100% of patients with SLE suffer pathological kidney damage^37^. Thus, evaluations of kidney pathology and renal function are crucial for the assessment of SLE. In this study, serum creatinine was used to assess renal function. We found that 4 weeks after ALD-DNA immunisation, serum creatinine levels were increased markedly in the ALD-DNA group and peaked at the 8th week. However, the rise was reduced significantly with AZM treatment. Pathologically, ALD-DNA induced relatively high inflammatory infiltration, renal tubular swelling, interstitial congestion, glomerulofibrosis, IgG deposition and glomerular basement membrane thickening, which were relieved by AZM. The above data sufficiently demonstrated “ALD-DNA-induced lupus” in terms of autoimmune antibodies, renal function and kidney pathology. Moreover, AZM showed an exciting therapeutic effect on ALD-DNA-induced lupus in vivo for the first time. Since occasional AZM-induced impaired hepatic function was reported previously^38^, we tested the levels of AST and ALT at the end point. Fortunately, no significant changes were detected.

AZM was first reported to regulate macrophage polarisation in vivo and in vitro in 2008 (Murphy et al.^39^). However, the role of AZM in SLE remains unclear. In our previous study, we indicated that AZM could promote M2 polarisation in lupus conditions in vitro. In the current study, we further investigated whether AZM can induce M2 polarisation in lupus mice in vivo for the first time. As the spleen is the largest immune organ containing a massive population of macrophages^40^, we assessed macrophage polarisation in the spleen. The results showed extremely more macrophage infiltration and M1 dominance in spleens from the ALD-DNA group than in those from the control group, which was largely in line with previous studies^12,41^. Notably, the administration of AZM significantly alleviated macrophage infiltration, and the proportion of M2 macrophages rebounded. To indicate the macrophage phenotypes were functional, we also analysed M1/M2-related genes in the kidneys because the kidney is the main target organ and very vulnerable in lupus^42,43^. ALD-DNA immunisation led to significant upregulation of M1-related genes and inhibition of M2-related genes. In contrast, M1-related genes were downregulated after AZM treatment, while M2-related genes were upregulated to varying degrees. All these data demonstrated that ALD-DNA played a distinctive role characterised by increased macrophage infiltration and M1 dominance, which are consistent with the M1 dominance in lupus reported by Orme and Mohan^44^. For the first time, we confirmed that AZM could promote M2 polarisation in lupus in vivo.

Considering that the ALD-DNA model was artificially induced to imitate lupus, further studies were performed in spontaneous murine lupus model mice. As a lupus-prone murine model, MRL-*Fas*^lpr^ mice showed higher levels of anti-dsDNA antibodies and serum creatinine than ALD-DNA-induced lupus mice, corresponding to early onset, rapid development and severe renal damage^45–47^. After AZM treatment, there was a slowdown in the upward trend of the anti-dsDNA antibody level, but the level continued to increase with time. The slowdown in this upward trend showed that AZM could alleviate the development of lupus in MRL-*Fas*^lpr^ mice but could not totally restrain it. As we discussed above, the dosage, delivery route and length of administration of the treatment should be investigated further to improve the efficacy. The changes in pathology and serum creatinine levels also indicated that AZM effectively reversed kidney damage. Next, we explored whether AZM could induce M2 polarisation in vivo in MRL-*Fas*^lpr^ mice. As expected, the number of infiltrated macrophages in the spleen was decreased after AZM treatment, with a remarkably higher proportion of M2 macrophages. The assessment of M1/M2-related genes also showed significant downregulation of M1-related genes and upregulation of M2-related genes after AZM treatment. All these data suggested again that M1 macrophages function in a “destroyer” role in the development of SLE, while M2 macrophages play a “healer” role. Overall, we are convinced that AZM can promote M2 polarisation in lupus in vivo, which ultimately alleviates the symptoms of SLE.

Taken together, our results demonstrated for the first time that AZM could alleviate lupus by promoting M2 polarisation in vivo. Our findings further confirmed the efficacy of AZM was mediated via macrophage regulation and may hopefully further expand the clinical application of AZM.

## Materials and methods

### Mice

Eight-week-old female BALB/c mice and 4-week-old female lupus-prone MRL-*Fas*^lpr^ mice were purchased from Shanghai SLAC Laboratory Animal Co., Ltd. (SLAC, Shanghai, China) and housed in pathogen-free housing at Fudan University. All animal experiments were ethically approved by the Animal Care and Use Committee of Fudan University.

### Generation of SLE murine models and tissue preparation

ALD-DNA was prepared as previously described^21^. As a pilot experiment, twelve female BALB/c mice were randomly divided into three groups (*n* = 4): the normal control group (NC group), ALD-DNA-induced mouse group (ALD-DNA group) and azithromycin-treated mouse group (ALD-DNA + AZM group). The mice in the ALD-DNA group were subcutaneously injected under the cervical skin with ALD-DNA (50 μg/mouse) plus complete Freund’s adjuvant (Sigma-Aldrich, St. Louis, MO, USA) at week 0, followed by two immunisations of ALD-DNA (50 μg/mouse) with incomplete Freund’s adjuvant (Sigma-Aldrich) at weeks 2 and 4, as described previously^22–24^. The mice in the AZM group were treated with AZM (150 mg/kg, Pfizer, New York, NY, USA) by intraperitoneal injection (two times per week) while receiving ALD-DNA immunisation. Mouse serum samples were obtained retro-orbitally every 4 weeks. All mice were killed at the 12th week.

Eight female lupus-prone MRL-*Fas*^lpr^ mice were randomly divided into two groups (*n* = 4): the MRL-*Fas*^lpr^ group and the MRL-*Fas*^lpr^ + AZM group. The mice in the treatment group were treated with AZM (150 mg/kg, Pfizer) by intraperitoneal injection (two times per week). Serum samples were collected every 2 weeks. All mice were killed at the 8th week.

### Anti-dsDNA antibodies

Serum anti-dsDNA antibody levels were determined with a mouse anti-dsDNA ELISA kit (Shibayagi, Gunma, Japan) according to the manufacturer’s specifications. Briefly, standards or samples were incubated in dsDNA-coated wells. After 2 h of incubation, a horseradish peroxidase (HRP)-labelled goat anti-mouse IgG antibody was added and incubated for 1 h. After washing, a chromogen was added to each well, and colour developed. The reaction was stopped by the addition of an acidic solution, and the absorbance was measured at 450 nm.

### Histology

Kidneys were isolated, fixed in 4% paraformaldehyde (Sangon Biotech, Shanghai, China) overnight, washed in phosphate-buffered saline (PBS) (Thermo Fisher Scientific, Waltham, MD, USA), embedded in paraffin, and then stained with H&E, Masson stain or PAS following standard procedures. Images were acquired using a light microscope (Zeiss, Oberkochen, Germany). Histology was assessed by a pathologist blinded to the experimental conditions.

### IgG deposition

Sections of kidneys were incubated with goat anti-mouse IgG Cy3 (Jackson ImmunoResearch, West Grove, PA, USA, catalogue number 115-165-146) for 1 h in the dark and mounted with Prolong gold antifade reagent (Thermo Fisher Scientific). Immune complex deposits were observed by fluorescence microscopy (Zeiss).

### Flow cytometry

Murine splenic tissues were surgically resected, ground and filtered to obtain single-cell suspensions. Splenic macrophages were gated by using a fluorescein isothiocyanate (FITC)-conjugated anti-mouse F4/80 antibody (eBioscience, San Diego, CA, USA, catalogue number 11-4801-82) on the basis of the side scatter parameter. The expression of CD80 (phycoerythrin-conjugated, eBioscience, catalogue number 12-0801-82) and CD206 (allophycocyanin-conjugated, eBioscience, catalogue number 17-2061-82) was measured in mouse macrophages. Flow cytometry data were obtained on a BD FACSCanto flow cytometer (BD Bioscience, Franklin Lake, NJ, USA) and analysed with FlowJo software (TreeStar, Inc., Ashland, OR, USA).

### Quantitative real-time polymerase chain reaction

Total RNA was extracted from the kidneys with TRIzol Reagent (Thermo Fisher Scientific) according to the manufacturer’s instructions. cDNA was obtained using a PrimeScript RT reagent kit (Takara, Otsu, Japan). The expression levels of IL-1β, IL-6, TNF-α, iNOS, CD86, TLR2, TLR4, TGF-β, Arg-1, Fizz-1, Ym-1 and CD206 were quantified by real-time polymerase chain reaction (PCR) using TB Green Premix Ex Taq II (Takara) and analysed on a QuantStudio 6 Flex Real-Time PCR system (ABI, Foster City, USA). All gene expression levels are presented as 2^−ΔΔCt^ calculations, using glyceraldehyde-3-phosphate dehydrogenase (GAPDH) as the housekeeping gene. The primer sequences used in this study are shown in Supplementary Table 1.

### Statistical analysis

All numeric data are expressed as the mean ± standard deviation and were assessed using SPSS 20.0 software (SPSS Inc., Chicago, IL, USA). Homogeneity of variance was assessed by the Friedman test. For data with equal variances, statistical analyses for comparisons between two groups were performed by Student’s *t* test (two-tailed), and multiple-group comparisons were performed by one-way ANOVA combined with the LSD post hoc test. For data with unequal variances, Welch’s *t* test and Welch’s ANOVA were used. Statistical significance was determined at *p* < 0.05.

## Supplementary information

supplementary Table 1
