# Supplementary material for: Azithromycin alleviates systemic lupus erythematosus via the promotion of M2 polarisation in lupus mice
Source: Cell Death Discov. 2021 Apr 16;7:82. doi: 10.1038/s41420-021-00466-4 (PMC8050155; doi:10.1038/s41420-021-00466-4)
Supplement: Supplementary file 1 — supplementary Table 1 [file 41420_2021_466_MOESM1_ESM.docx]

Supplementary Table 1. Primer sequences for quantitative real-time PCR

| Gene | Sense primer | Antisense primer |
| --- | --- | --- |
| Mouse GAPDH | GAGCCAAACGGGTCATCATCT | GAGGGGCCATCCACAGTCTT |
| Mouse IL-1β | GAAATGCCACCTTTTGACAGTG | TGGATGCTCTCATCAGGACAG |
| Mouse IL-6 | ACAACCACGGCCTTCCCTACTT | CACGATTTCCCAGAGAACATGTG |
| Mouse CD86 | TGTTTCCGTGGAGACGAAG | TTGAGCCTTTGTAAATGGGCA |
| Mouse iNOS | GTTCTCAGCCCAACAATACAAGA | GTGGACGGGTCGATGTCAC |
| Mouse TNF-α | AAGCCTGTAGCCCACGTCGTA | GGCACCACTAGTTGGTTGTCTTTG |
| Mouse TLR-2 | GCAAACGCTGTTCTGCTCAG | AGGCGTCTCCCTCTATTGTATT |
| Mouse TLR-4 | ATGGCATGGCTTACACCACC | GAGGCCAATTTTGTCTCCACA |
| Mouse TGF-β | CTCCCGTGGCTTCTAGTGC | GCCTTAGTTTGGACAGGATCTG |
| Mouse CD206 | CAAGGAAGGTTGGCATTTGT | CCTTTCAGTCCTTTGCAAGC |
| Mouse Arg-1 | CAAGACAGGGCTCCTTTCAG | GTAGTCAGTCCCTGGCTTATGG |
| Mouse Ym-1 | AGAAGGGAGTTTCAAACCTGGT | GTCTTGCTCATGTGTGTAAGTCA |
| Mouse Fizz-1 | CCTGCTGGGATGACTGCTACT | AGATCCACAGGCAAAGCCAC |
